# Bioclimatic Analysis and Spatial Distribution of Livestock Fascioliasis in Iran

**Published:** 2019

**Authors:** Mohamad GHANIMATDAN, Abdolali CHALECHALE, Farid REZAEI, Mohamad Bagher ROKNI, Seyed Reza SHAHROKHI

**Affiliations:** 1. Department of Pathobiology, Faculty of Veterinary Medicine, Razi University, Kermanshah, Iran; 2. Department of Medical Parasitology and Mycology, School of Public Health, Tehran University of Medical Sciences, Tehran, Iran; 3. Department of Medical Parasitology and Mycology, School of Medicine, Shahid Beheshti University of Medical Sciences, Tehran, Iran

**Keywords:** Fascioliasis, Bioclimatic analysis, Spatial distribution, Risk map, Iran

## Abstract

**Background:**

This study aimed to investigate the prevalence of fascioliasis and to perform a climatological analysis of different regions of Iran based on the current situation of the parasite and its intermediate host using Geographical Information System (GIS).

**Methods:**

Meteorological data were obtained from Iran Meteorological Organization. Risk map of fascioliasis transmission was prepared based on this data and using forecasting indices. Further, the number of fascioliasis cases from 31 provinces reported to the Iran Veterinary Organization were collected and prevalence maps of livestock fascioliasis were drawn.

**Results:**

The main risk hotspots were found in Northern provinces like Golestan, Mazandaran and Gilan as well as some Southern provinces such as Kohgiluyeh and Boyer-Ahmad and Chaharmahal and Bakhtiari and Fars, which have ideal conditions for completion of the parasite life cycle. Moreover, Gilan Province with 10.83% had the highest rate of fascioliasis infection in slaughtered animal.

**Conclusion:**

Iran is one of the most important foci of fascioliasis globally. Several provinces of Iran have appropriate conditions for evolution of parasite life cycle and presence of its intermediate host. These regions require special attention and serious determination in order to control fascioliasis in human and animals.

## Introduction

Fascioliasis or distomiasis is caused by two trematodes, *Fasciola hepatica* and *F. gigantica*, belonging to *Fasciola* genus. Both species have been reported worldwide, but infections mainly occur in tropical regions of Africa, South and East Asia and Middle East. Overall, 2.4–17 million people are infected with fascioliasis, and the number of people at risk of infection is 180 million people globally (1). The humans and at least 46 mammalian species have been identified as the final hosts of *Fasciola*. However, the only intermediate host of this parasite is Lymnaeidae (1). In most parts of the world, including Asia, Europe and Africa, the intermediate host snail of *F. hepatica* is *Lymnaea truncatula*. The most important intermediate host of *F. gigantica* is *L. auricularia* or *L. gedrosiana* (2, 3).

Both *F. hepatica and F. gigantica* have been reported from sheep, goats, cattle (4–6), buffalo (7), camels (8), horses (9) and wild boar (10) in Iran. Two major epidemics and several interepidemics occurred during the 1980s, and 1990s in Gilan Province, northern Iran (11, 12).

From the egg disposal stage to its development into cercaria is a time when the parasite is sensitive to different climatic changes. Environmental conditions such as temperature, precipitation, humidity, soil conditions, and solar radiation directly affect the survival of *F. hepatica* in the environment and development of parasite as well as survival, growth and proliferation of intermediate host of parasite (13, 14).

We performed a spatial analysis on the prevalence of fascioliasis in the ruminants, prepared the prevalence maps, analyzed the meteorological and environmental factors affecting the development of parasite and its snail host and presented the risk maps of disease in Iran.

## Materials and Methods

### Data collection

The meteorological data of a ten-yr period (2007–2017) in 31 provinces of Iran were obtained from Iran Meteorology Organization (www.irimo.ir). The slaughterhouses report over a six-yr period (2012–2017) as well as the statistics of livers infected with fascioliasis in sheep, goat and cow from all slaughterhouses of the country were collected in cooperation with Iran Veterinary Organization (www.ivo.ir).

The collected data fended into Microsoft Excel 2010, and the prevalence rate of monthly fascioliasis at slaughterhouses around the country was obtained by the following formula. In this formula, the numerator (Pi) is the number of livers infected with fascioliasis and the denominator (Ps) is the number of slaughtered livestock.

$$P=\frac{P_{i}}{P_{s}}$$

The meteorological data and prevalence statistics were transferred to the ArcGIS 10.3 database as attribute table and attached to Iran’s map.

### Climatic analyses

#### Classification of country

The relative effects of five variables, including average monthly temperature, monthly precipitation, humidity, elevation and slope on the risk of fascioliasis transmission based on the physiology of parasite and intermediate host snail were investigated (Table 1) (13–19). Accordingly, the relative risk of fascioliasis transmission was classified into three classes, including “suitable for disease transmission” (C1 regions), “limited disease transmission” (C2 regions) and “minimal disease transmission” (C3 regions).

**Table 1:** Effect of environmental and meteorological conditions on the transmission risk of fascioliasis

***Variables***	***Relative risk***		
	***Suitable (C1)***	***Limited (C2)***	***Minimal (C3)***
Temperature (°C)	15–25	10–15 or 25–30	<10 or >30
Precipitation (mm/year)	>1500	700–1500	<700
Elevation (m)	<500	500–1000	>1000
Relative humidity (%)	>60	30–60	<30
Slope (Degree)	<10	10–15	>15

### Ollerenshaw index

Using Ollerenshaw index, the relative risk of fascioliasis transmission in various regions was calculated. Different regions of the country were classified into four groups according to obtained value from the following equation of this index (Mt). If Mt is less than 300, the transmission risk of fascioliasis is low (C4), if it is 300–400, the transmission risk is not at the limit of permanent transmission of the disease (C3), if it is 400–474, fascioliasis is prevalent in that region (C2) and if it is more than 474, the disease can be endemic in that region (C1) (17, 18).

$$\text{Mt}=\text{n}\left(\frac{\text{R}}{25.4}-\frac{\text{PET}}{25.4}+5\right)$$

Where (Mt) is Ollerenshaw index, (n) is number of days with rainfall, (R) is monthly Precipitation and (PET) is potential evapotranspiration

PET level is influenced by different climatic factors such as maximum monthly temperature, air humidity and daily solar radiation, and is calculated by various methods. In this study, FAO Penman-Monteith formula (20), presented below, was used to compute PET.

$$\text{PET}=0.0023\times 0.408\times \text{Ra}\left(\frac{{\text{T}}_{\text{max}}-{\text{T}}_{\text{min}}}{2}+17.8\right)\sqrt{{\text{T}}_{\text{max}}-{\text{T}}_{\text{min}}}$$

Where (PET) is potential evapotranspiration, (Ra) is net radiation at the crop surface, (Tmax) is maximum monthly temperatures and (Tmin) is minimum monthly temperatures.

### Growing Degree Days (GDD) and Malone index

The growth rate of many living beings is first controlled by temperature. A temperature in which maximum growth occurs is called optimal temperature. With a rise in temperature to above optimal temperature, the growth rate is reduced. The GDD index calculates the ideal temperature required for the development of living things. In the equation of this index, Tbase or minimum temperature required for evolution of living beings and Tm or monthly average temperature are variables. This Tbase has been reported to be 10 °C for *F. hepatica* and 16 °C for *F. gigantica* (19). The equation for calculation of GDD is as follows:

$$\int ({\text{T}}_{\text{m}}-{\text{T}}_{\text{base}})\text{dt}$$

The Malone depends on the numerical value of GDD, monthly precipitation and PET rate (21). The formula of this index is presented below.

$$\begin{array}{ll} \text{Malone}\;\text{Index} & =\left[\text{GDD}\times 30,\text{if}(\text{R}-0.8\text{PET}>0)\right]\\ & +\left[\left(\text{GDD}\times 6)\times \left(\frac{\text{R}-\text{PET}}{25}\right),\text{if}(\text{R}-\text{PET}>0\right)\right] \end{array}$$

In months in which R-0.8 PET>0 is established, there is adequate humidity for the parasite to continue its life cycle in most days of the month in 2.5 cm above land surface. Therefore, GDD is multiplied by total days of the month. Accordingly, Iran was divided into four regions. If Malone index is less than 600, there is no risk of fascioliasis in that region (C4), and if it is 600–1500, there is low possibility of occurrence of the disease (C3). Regions with Malone index of 1500–3000 have a higher risk of the continuation of parasite life cycle and incidence of fascioliasis (C2). The regions with Malone index of over 3000 have a very high risk of fascioliasis transmission (C1).

### Data analysis and mapping

A set of 19 raster and vector layers, including basic geographic data (administrative division map of Iran), bioclimatic datasets (average monthly temperature, monthly precipitation, relative humidity, elevation and slope) and prevalence data (georeferenced prevalence of infected livestock) were prepared. Prevalence maps, risk maps and Standard deviation ellipse is drawn using spatial analyst (Kriging interpolation and Standard deviation ellipse method). The statistical difference between the prevalence of disease, year and province was analyzed by SPSS Ver. 18 (Chicago, IL, USA) using one-way ANOVA. Pearson correlation coefficient was used to study the correlation among the prevalence of animal fascioliasis, altitude, annual precipitation, mean humidity, mean temperature and results of Ollerenshaw and Malone indices.

## Results

### Analysis of fascioliasis prevalence and preparation of prevalence maps

According to the slaughterhouse statistics, from 100594543 slaughtered animals reported after meat inspection stage, 2923146 livers (2.9%) were infected with fascioliasis. The prevalence rate of fascioliasis in Iran during this period was calculated as 2.87% for the slaughtered sheep, 1.99% for the slaughtered goats and 4.55% for the slaughtered cows.

The prevalence rate of fascioliasis in each province was computed (Table 2) and analyzed by one-way ANOVA in different provinces. Gilan (10.83%) and Kohgiluyeh and Boyer Ahmad (5.52%) provinces had the highest rate of infection, and Semnan (0.37%) and North Khorasan (0.53%) provinces had the lowest prevalence of animal fascioliasis (*P*=0.001). Although the prevalence of fascioliasis was higher in 2016 than other years (Table 3), the difference between prevalence of disease in various years was only significant on fascioliasis among sheep (*P*=0.04).

**Table 2:** Prevalence of fascioliasis in Iran

***No.***	***State***	***Sheep fascioliasis (%)***	***Goat fascioliasis (%)***	***Cow fascioliasis (%)***	***Total fascioliasis (%)***
1	Alborz	0.98	3.03	3.05	1.49
2	Markazi	1.23	0.71	2.45	1.23
3	Mazandaran	4.86	3.09	5.05	4.32
4	Khuzestan	2.28	2.53	9.73	3.21
5	Bushehr	2.75	0.71	1.45	1.31
6	Kermanshah	1.43	2.23	3.89	2.11
7	Ilam	1.31	1.29	5.39	1.87
8	Lorestan	2.79	1.84	9.83	3.85
9	Kurdistan	5.26	2.76	5.71	5
10	Isfahan	1.78	1.29	2.58	1.6
11	Chaharmahal and bakhtiari	1.45	2.7	9.29	2.39
12	Gilan	11.95	5.1	15.18	10.83
13	Hamadan	2	1.61	2.45	2.04
14	Tehran	1.76	3.07	8.63	2.68
15	Qazvin	1.52	0.95	1.22	1.33
16	Semnan	0.3	0.31	1.41	0.37
17	Hormozgan	3.78	1.72	2.74	2.48
18	Kerman	0.9	1.05	10.11	1.07
19	South Khorasan	0.7	0.7	0.36	0.65
20	Razavi Khorasan	0.83	0.3	1.92	0.68
21	North Khorasan	0.77	0.13	0.84	0.53
22	Golestan	1.65	1.71	3.33	1.86
23	Fars	0.6	0.43	1.29	0.57
24	Kohgiluyeh and Boyer-ahmad	7.28	4.03	10.37	5.52
25	Ardabil	6.06	1.29	4.19	5.29
26	West Azerbaijan	1.86	1.84	4.65	3.10
27	East Azerbaijan	3.97	2.91	2.72	3.63
28	Zanjan	6.92	5.62	2.24	4.31
29	Yazd	0.65	0.64	2.21	0.7
30	Zahedan	7.25	4.88	2.82	2.9
31	Qom	2.14	1.33	4.06	2.06
-	Total	2.87	1.99	4.55	2.9

**Table 3:** Prevalence of fascioliasis during 2012–2017 in Iran

***No.***	***Year***	***Cow fascioliasis (%)***	***Goat fascioliasis (%)***	***Sheep fascioliasis (%)***
1	2012	2.45	1.46	3.65
2	2013	2.85	1.94	4.37
3	2014	2.91	2.07	4.67
4	2015	2.89	1.96	4.55
5	2016	3.11	2.23	4.9
6	2017	3.02	2.3	5.18
	Total	2.87	1.99	4.55

**Fig. 1:**
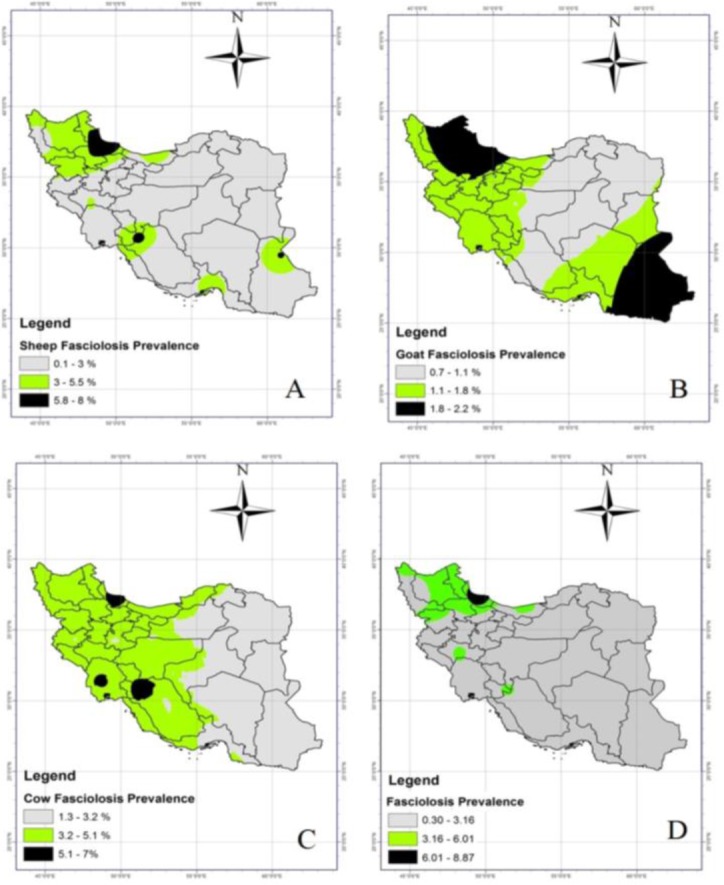
Prevalence map of fascioliasis in Iran. A: Sheep, B: Goat, C: Cow, and D: Total fascioliasis

Spatial analysis and preparation of prevalence maps of fascioliasis showed that three major hotspots of fascioliasis in sheep in Iran were in northern region, including Gilan and Ardabil provinces, southern region, including Fars, Kohgiluyeh and Boyer Ahmad and Chaharmahal and Bakhtiari provinces and southeast region, including Sistan and Baluchistan Province. The southeastern region and northern region were the major hotspots of goat fascioliasis prevalence in Iran. Moreover, goat slaughter was reported to be more in the eastern and southeast provinces than other provinces. The major hotspots of fascioliasis prevalence in cows included southern region, including Fars, Kohgiluyeh and Boyer Ahmad, Chaharmahal and Bakhtiari and Khuzestan provinces and Gilan Province in the north of Iran (Fig. 1).

**Fig. 2:**
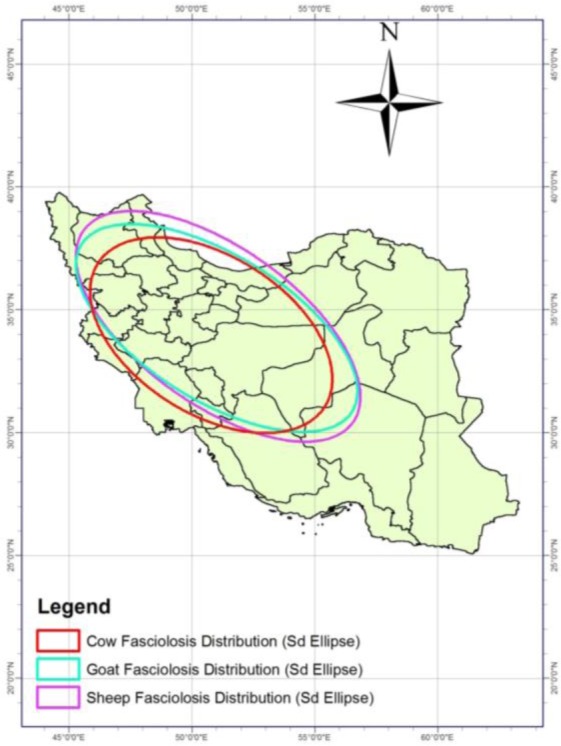
SD ellipse of prevalence of fascioliasis in Iran

Standard deviation ellipse indicated which the highest dispersion of prevalence of fascioliasis for all three animals, cow, goat and sheep in the north and northwest regions of Iran (Fig. 2).

### Climatic analysis

The appropriate average annual temperature for the intermediate host snails of *Fasciola* is 15–25 °C. For the intermediate host survival a minimum temperature of 10 °C and maximum between 18 and 27 °C is required (22). Ardabil (10.66 °C), Chaharmahal and Bakhtiari (11.77 °C) and West Azerbaijan (11.88 °C) were the coldest provinces, and Hormozgan (27.23 °C), Khuzestan (26.62 °C) and Bushehr (25.76 °C) were the hottest provinces based on average annual temperature in this period. There was no significant correlation between temperature rise and fascioliasis prevalence (*P*=0.106).

Rainfall conditions in most regions of Iran, except Northern provinces, are not in favor of the growth and proliferation of host snail of *Fasciola*. However, seasonal rains in winter and spring and relatively good humidity in some regions have provided the conditions for the growth and proliferation of snails.

**Fig. 3:**
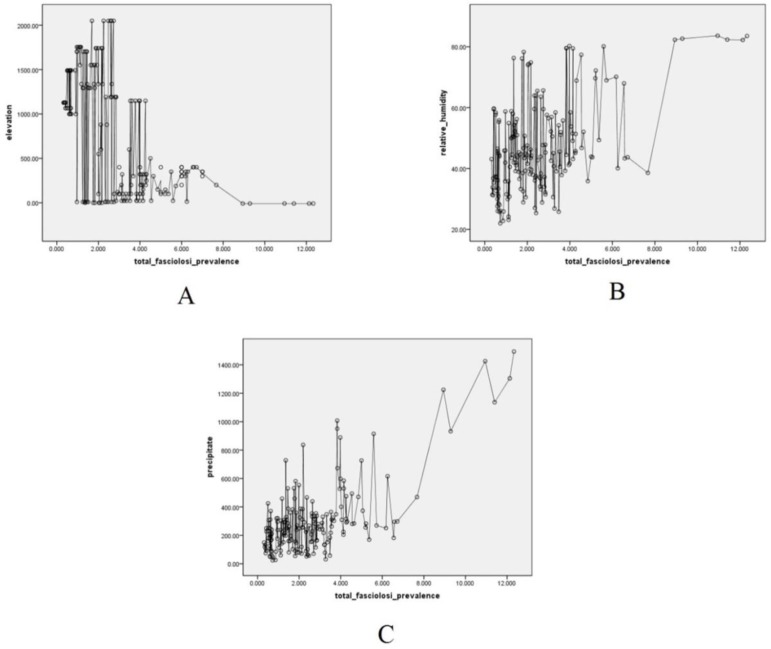
Correlation between environmental parameters and prevalence of fascioliasis in Iran. A: Elevation, B: Humidity and C: Rainfall

The results of Pearson correlation showed a positive correlation between increased relative humidity and increased prevalence rate of fascioliasis (Pearson Correlation = 0.685, *P*=0.001) and a reverse correlation between altitude above sea level and increased prevalence of fascioliasis (Pearson Correlation= −0.241, *P*=0.001) (Fig. 3).

**Fig. 4:**
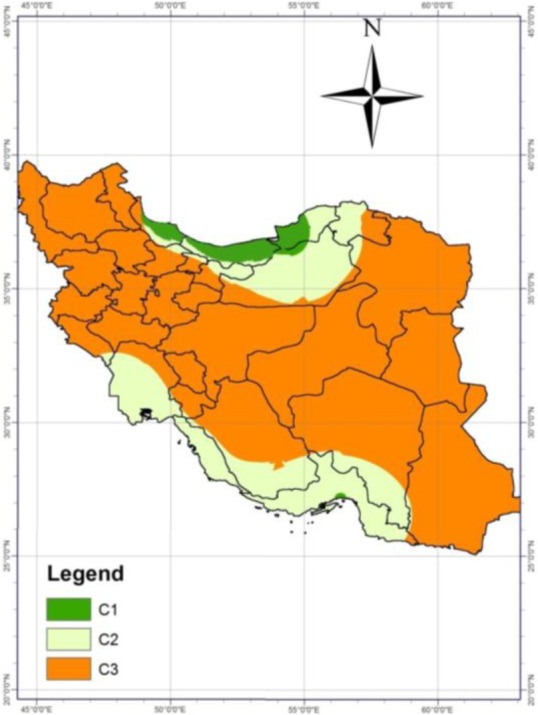
Risk map of fascioliasis based on environmental and meteorological conditions

The risk map of fascioliasis prevalence in the country was prepared by combining the raster maps obtained from environmental and meteorological conditions (Fig. 4). In this risk map, the main risk centers for prevalence of fascioliasis were the north and south of Iran.

Mazandaran, Gilan and Golestan provinces were located in C1, which have the highest possibility for the presence of Lymnaeidae and disease transmission in these regions. Other northern provinces like Ardabil and north Semnan as well as southern provinces such as Hormozgan, Bushehr, Fars and Khuzestan were found to be in C2, where the risk of disease transmission is feasible. The possibility of disease transmission was very low in other regions of the country.

### Ollerenshaw index

This index is influenced by two parameters, monthly precipitation and PET. The findings of Pearson correlation test showed a positive correlation between Ollerenshaw index calculated in different regions and prevalence rate of animal fascioliasis (Pearson Correlation=0.530, *P*=0.001).

**Fig. 5:**
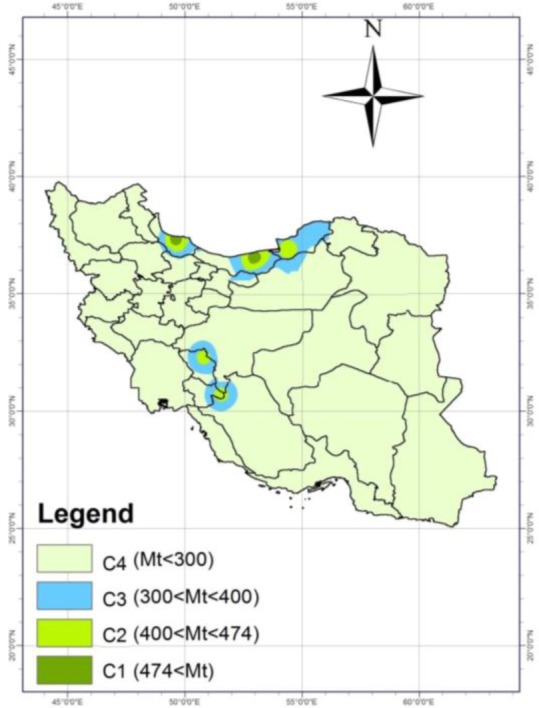
Risk map of fascioliasis based on Ollerenshaw index

The risk map of Ollerenshaw index in Iran showed that the main hotspots of this disease (C1) were only Mazandaran and Gilan provinces. Golestan, Chaharmahal and Bakhtiari and Kohgiluyeh and Boyer Ahmad provinces were in C2 and C3 regions of this index, expected to have snails, more transmission of disease and more prevalence of fascioliasis. More than 90% of regions (the rest of regions except the mentioned provinces) were in C4 regions, where there is no risk of permanent transmission of disease (Fig. 5).

### Malone index

**Fig. 6:**
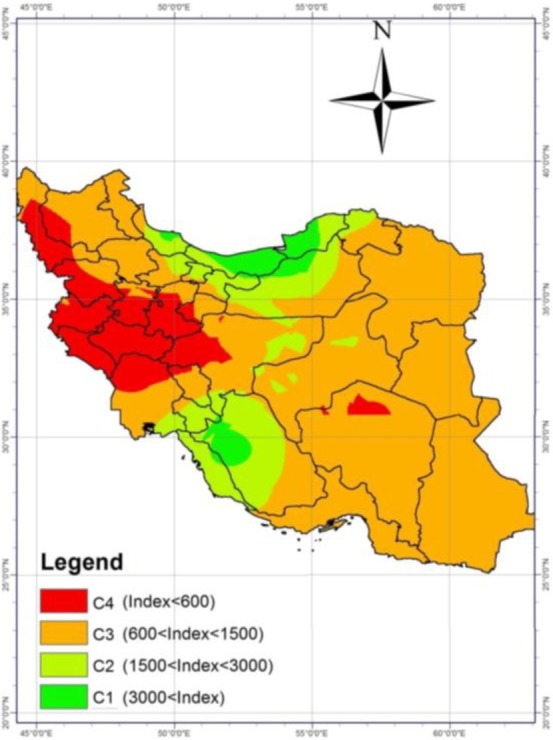
Risk map of fascioliasis based on Malone index

According to this index, the geographical regions were divided into four regions (Fig. 6).

The northern regions of the country, including Golestan, Mazandaran, Gilan and northern part of Semnan, Tehran, Alborz and Qazvin provinces as well as southern provinces, including Fars, Kohgiluyeh and Boyer Ahmad and Chaharmahal and Bakhtiari were categorized into C1 and C2 regions and were considered the main hotspots for the risk of prevalence of fascioliasis in Iran. In other regions, the possibility of occurrence of this disease was less than the mentioned centers. There was a positive correlation between the numerical value of this index and prevalence of fascioliasis in slaughtered animals (Pearson Correlation=0.582, *P*=0.000).

## Discussion

Maps predicting fascioliasis risk have been created for several endemic areas in Cambodia created a map based on proximity to rivers, density of livestock and ecological parameters (23). Other studies in east Africa were based on moisture and temperature (21, 24). An inverse correlation between *F. hepatica* infection rate and the altitude observed in cattle in Brazil (25). Greatest risk for *F. hepatica* occurrence was extended rainfall and soil moisture, as the risk of fascioliasis diminished in the driest and cooler areas of East Africa (21). A risk model in Ireland show that the most significant predictors were the total rainfall and total wet days (26).

Coastal regions of the Caspian Sea, including Gilan and Mazandaran provinces and, to a lesser extent, Golestan Province had favorable conditions for the completion of the life cycle of parasite. The studies conducted on the prevalence of animal fascioliasis in Iran have mainly been performed through meat inspection on the sheep, goats and cows slaughtered in different slaughterhouses of the country and have reported infection with fascioliasis in various regions. Gilan (27), Mazandaran (28), Kohgiluyeh and Boyer Ahmad (29), Kerman (30) and Lorestan (31, 32) provinces have the highest prevalence of this disease (35.5%, 20.14%, 11.15%, 9.26% and 6.3%, respectively).

The frequent rainfall in northern provinces as well as Kohgiluyeh and Boyer Ahmad, Chaharmahal and Bakhtiari and Fars provinces has provided the ground for presence and proliferation of intermediate host snail of *F. hepatica*. Another important point is rainfall in the hot months of the year (from late spring to late summer) in the northern provinces of the country.

The risk maps and prevalence maps prepared in this study showed that Guilan Province is the most important hotspot of fascioliasis in Iran. This province has been the focus of previous studies due to a large epidemic in 1989 and another one in 1999 (2). The largest outbreak of fascioliasis in the world and the first epidemic recorded in Iran in 1989 occurred in Gilan Province, and according to the estimates, over 7000–10000 people were infected in the 1989 epidemic and thousands in 1999 epidemic (12, 33–36). During the epidemic course, positive rate for fascioliasis was 50% there. More than 70% of fascioliasis reports in these years were in Anzali City. In Anzali and Rasht, both located in the endemic region of this disease in Gilan Province, infection with fascioliasis was associated with oral aquatic plants probably infected with metacercariae (37). Mazandaran in north of Iran is another important hotspot of fascioliasis. During 1999–2002, 107 cases of human infection with fascioliasis have been reported in this province (38).

In 2000, a small outbreak with 17 cases of fascioliasis, occurred in the west of Iran in Kangavar City, Kermanshah Province, western Iran. The possible source of this infection was watercress infected with *Fasciola* metacercariae, and infection with fascioliasis was prevalent in 1.5% of cows in that region (39).

Human studies have been mostly done on diagnosis of serum *Fasciola* in the blood sample of healthy people. In a study in Tehran on the patients from different provinces referred to the School of Public Health, Tehran University of Medical Sciences due to suspicious symptoms of fascioliasis showed that 24.8% of them had positive titer of fascioliasis (40). Studies on the serum prevalence of healthy people in other regions have reported less than 2% of participants had positive titer of fascioliasis. The highest prevalence of serum fascioliasis (1.7%) (41) was reported in Kohgiluyeh and Boyerahmad province, followed by Isfahan (1.7%) (42) and Ardabil (1.56%) (43) provinces.

A descriptive research studied and counted the snails of Mazandaran in 6 points. The population peak of *L. truncatula* and *L. auricularia* was found to be in July and peak of human fascioliasis cases reported to the health centers was late winter. The three-month development of parasite in snail and three-month incubation period indicate that the season of transmission is late fall. The comparison of human and animal cases shows a peak of infection in slaughterhouses, both in cows or sheep, which is in early spring (1 or 2 months after the peak of human cases) (44).

## Conclusion

Geostatistical methods in predicting the possible transmission of the disease, results of statistical analyses of the animal fascioliasis prevalence, various studies on the prevalence rate of fascioliasis in human and animal as well as presence of the host snails of the parasite in many areas of the country and history of occurrence of several epidemics show that Iran is one of the most important hotspots of fascioliasis around the world. With an increase in precipitation in the hot seasons, the population of host snails of *F. hepatica* is increased dramatically, and possibility of the occurrence of fascioliasis, as it occurred in the epidemic of Gilan Province, is increased. The damages resulting from the disease in human and animals are noticeable, and infection with disease is one of the problems of traditional and modern livestock breeding. Northern areas like Gilan, Mazandaran and Golestan provinces as well as Kohgiluyeh and Boyerahmad, Chaharmahal and Bakhtiari and Fars provinces are favorable regions for the growth and proliferation of parasite and its intermediate host snail, and prevalence of disease in human and animal in these provinces requires more supervision and control. Using GIS and further studies, more accurate climatic analyses can be performed on the relationship of different environmental factors with incidence of fascioliasis in potential hotspots of the disease in order to identify the important points, risk factors of occurrence and prevalence maps of the disease in these provinces.
